# Spontaneous pineal apoplexy in a pineal parenchymal tumor of intermediate differentiation

**DOI:** 10.7497/j.issn.2095-3941.2013.01.007

**Published:** 2013-03

**Authors:** Ching-Chun Wang, Jennifer Turner, Timothy Steel

**Affiliations:** 1 Nepean Hospital, Kingswood, NSW 2747, Australia;; 2 St. Vincent’s Hospital, Darlinghurst, NSW 2010, Australia

**Keywords:** Apoplexy, pineal parenchymal tumor, obstructive hydrocephalus, pineal gland, Parinaud’s syndrome

## Abstract

Pineal apoplexy is a rare clinical presentation of pineal parenchymal tumors. We report the curative treatment of a case of pineal parenchymal tumor of intermediate differentiation with spontaneous apoplectic hemorrhage. This case is shown through computed tomography and magnetic resonance imaging of the brain, and is confirmed via histopathological studies. Recurrent upward gaze paresis was observed after the stereotactic biopsy. The paresis required an expeditious tumor resection. The mechanism of the pineal apoplectic hemorrhage remains unclear although it has been observed in different pineal region lesions. Clinical and radiological evidence of the cure 5 years post-surgery is available.

## Introduction

Pineal apoplexy is a very rare clinical syndrome, and is characterized by the acute worsening of headaches, nausea, vomiting, ataxia, and gaze paresis. The syndrome is secondary to an obstructive hydrocephalus and/or direct compression on the cerebellum or midbrain pretectum or tectum^1^^,^^2^. Spontaneous apoplectic hemorrhage in a pineal parenchymal tumor of intermediate differentiation (PPTID) as an initial clinical presentation was not found. However, pineal apoplexy has been related to heterogeneity of pineal pathologies^1^^-^^4^. This paper reports a case of PPTID with spontaneous pineal apoplexy.

## Case report

**Figure 1:**
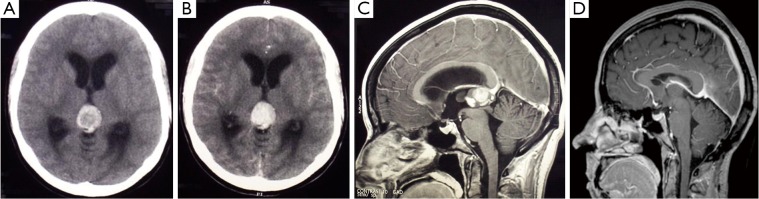
A. The plain CT scan shows a hyperdense pineal mass, which is suggestive of hemorrhage; B. The lesion is enhanced with contrast, and causes an obstructive hydrocephalus with the dilatation of the lateral ventricles. Sagittal T1-weighted gadolinium MRI scans; C. The pre-operative scan reveals a 2.0 cm × 2.5 cm pineal lesion with a well-defined margin, which compresses the cerebral aqueduct; D. The repeated scans at the 5-year follow-up shows no recurrence and complete tumor resection. The closeness of the internal cerebral vein and vein of Galen to the tumor should be noted. These veins remain obvious postoperatively. There is evidence of decompression of the ventricles and cerebral aqueduct.

A previously healthy 31-year-old woman presented sudden-onset headaches, vomiting, and photophobia. She experienced a progressively worsening headache for 5 days before the hospital presentation. The neurological examination revealed no abnormalities except for an upward conjugate gaze paresis (Parinaud’s syndrome). The computed tomography (CT) of the brain showed a 2.0 cm × 2.5 cm well-circumscribed hemorrhagic pineal lesion with contrast enhancement and an obstructive hydrocephalus. Magnetic resonance imaging (MRI) of the brain confirmed the hemorrhagic pineal lesion (**Figure 1**). A ventriculoperitoneal shunt was inserted to alleviate the effects of the obstructive hydrocephalus. Laboratory investigations conducted on the same day revealed within-normal ranges, including the coagulation studies. The tumor markers in the serum and cerebrospinal fluid (CSF), including the beta subunit of human chorionic gonadotropin and alpha-fetoprotein, were also normal. No malignant cells were found in the CSF cytology. The patient was then discharged from the hospital and was scheduled for a stereotactic biopsy of the pineal lesion 2 weeks later. Her upward conjugate gaze paresis was completely resolved on the second admission. She developed a recurrent upward conjugate gaze paresis, which was most likely caused by a post-biopsy intra-tumoral hemorrhage, a day after the biopsy. She then underwent craniotomy to have the lesion excised through the infratentorial supracerebellar approach. Complete macroscopic excision was achieved. The immediate post-operative CT scan showed complete tumor resection and resolution of the hydrocephalus. No post-operative complications were found. The patient was discharged 3 weeks later when the craniotomy showed no neurological deficits.

**Figure 2:**
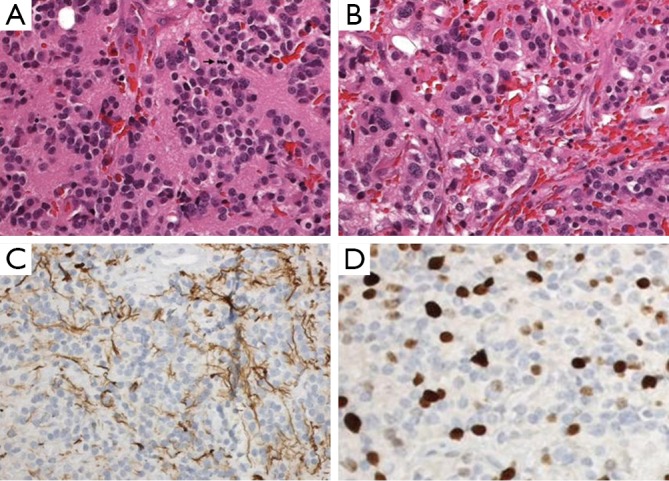
Pineal parenchymal tumor of intermediate differentiation. A. Area of tumors with uniform round nuclei and nucleus-free pineocytomatous rosettes containing fine fibrillary material. One mitosis is seen (arrow) (H&E staining, 400×); B. More cellular area with mild nuclear atypia and no rosettes (H&E staining, 400×); C. Moderate numbers of neurofilament protein expression among the tumor cells (400×); D. Several Ki-67 labeled nuclei of tumor cells (400×).

The histological studies revealed a moderate cellular tumor composed of small cells with round and generally uniform nuclei, fine chromatin, and small nucleoli. A few foci of cells with mild nuclear atypia were also found. Mitotic figures were rare (fewer than one per ten high-power field). Small pineocytomatous rosettes were focally found . Extensive hemorrhaging occurred in the adjacent granulation tissue, gliosis, and hemosiderin-laiden macrophages. The immunohistochemistry was positive for neurofilament, enolase, synaptophysin, and CD 56, but negative for NeuN . The Ki-67 proliferation index was 20% to 30% (**Figure 2**). The tumor was diagnosed as grade II PPTID based on the standards of World Health Organization (WHO).

No evidence of recurrence was found from the annual follow-up MRI studies. The patient also remained asymptomatic at the 5-year follow-up (**Figure 1**).

## Discussion

Pineal apoplexy, a poorly understood clinical syndrome, can be delineated as an acute neurologic deterioration caused by an abrupt expansion of a pineal lesion that is usually secondary to acute intratumoral hemorrhage^1^^,^^2^. The association of the pineal apoplectic hemorrhage with anticoagulant therapy and ventriculoperitoneal shunt placement has been reported, although the pathophysiology of the pineal apoplectic hemorrhage remains unclear^3^^,^^5^. A review of literature only yields 3 cases of pineal apoplexy associated with pineal parenchymal tumors (PPTs) (**Table 1**)^4^^,^^5^. Headaches and gaze paresis are the most common clinical symptoms of the pineal apoplectic syndrome as reported in more than 74% of affected individuals^2^. Nausea, vomiting, syncope, and ataxia are also found in more than 20% of the cases^2^.

**Table 1:** Reports of pineal apoplexy in association with pineal parenchymal tumors

Series (reference)	Ages (year)/sex	Clinical symptoms/signs	Predisposing factor	CSF tumor markers/cytology	Histopathological type	Time of histopathological diagnosis	Therapy	Radiological findings	Outcome
Steinbok *et al.*^4^	13/M	Headache, neck stiffness, lethargy, slow pupillary responses, and upward conjugate gaze paresis	Spontaneous	NR	Pineocytoma	Postmortem	VAS and radiation	Pneumoencephalography showed ventricular dilatation.	Death
								Ventriculography showed a third ventricular lesion.	
	33/F	Headache, optic atrophy with concentric visual field loss, and dilated and non-reactive pupils	Spontaneous	NR	Pineocytoma	Antemortem	VAS and radiation	Pneumoencephalography showed communicating hydrocephalus.	Death
								Radionuclide brain scan showed a pineal lesion.	
Matsumoto *et al.*^5^	58/M	Lethargy and conjugate upward gaze paresis	VPS insertion	CSF biomarkers (β-HCG, AFP, CEA, and PLAP): negative; CSF cytology: NR	Pineocytoma	Antemortem	VPS, EVD, surgical tumor resection (infratentorial supracerebellar approach), and the whole brain radiation (40 Gy to the ventricles and 10 Gy boost to the tumor bed)	CT showed obstructive hydrocephalus and intratumoral hemorrhage.	Recovery (at the 3-month follow-up)
								Angiography showed normal vasculature in the pineal region.	
								Post-EVD MRI showed reduction of ventricular size and intratumoral hemorrhage.	
Present case	31/F	Headache, vomiting, and conjugate upward gaze paresis	Spontaneous	CSF biomarkers (β-HCG and AFP): negative; CSF cytology: negative	Pineal parenchymal tumor of intermediate differentiation (WHO grade II)	Antemortem	VPS and surgical tumor resection (infratentorial supracerebellar approach)	CT and MRI showed obstructive hydrocephalus and intratumoral hemorrhage.	Recovery (at the 5-year follow-up)

AFP, alpha-fetoprotein; β-HCG, beta subunit of human chorionic gonadotropin; CEA, carcinoembryonic antigen; CSF, cerebrospinal fluid; CT, computed tomography; EVD, external ventricular drain; MRI, magnetic resonance imaging; NR, not recorded; PLAP, placental alkaline phosphatase; VPS: ventriculoperitoneal shunt; VAS, ventriculoatrial shunt; WHO, the World Health Organization.

PPTIDs were first introduced as a distinct pathological entity of PPTs in the central nervous system neoplasms by WHO in 2007. These PPTIDs were designated as grade II (low-grade; less than 6 mitoses and positive neurofilament stain) or  III tumors (high-grade; greater than or equal to 6 mitoses and negative for neurofilament stain)^6^. PPTIDs are very rare tumors, comprising of less than 0.1% of all primary central nervous system neoplasms^6^. The estimated recurrences range from  26% (WHO grade II; median time to recurrence =5 years) to 56% (WHO grade III; mean time to recurrence =1.3 years), and the 5-year overall survival rates vary between 39% (WHO grade III) and 74% (WHO grade II)^7^. The discrepancy is mainly because of the rarity of this entity and the subsequent paucity of data to establish a clinically relevant grading criterion. The optimal treatment for this entity remains elusive. Surgical tumor resection is generally recommended as an initial treatment for PPTs of all grades, with limited evidence supporting the application of adjuvant chemotherapy and radiation therapy for localized and low-grade PPTs^8^.

The precipitating factors of the initial pineal apoplexy in our patient could not be identified. Although known to be a safe and reliable procedure to obtain a histological diagnosis of pineal lesions^8^, stereotactic biopsy elicited a second pineal apoplexy that prompted an urgent surgical tumor resection. No adjuvant chemotherapy and radiation therapy were planned given the tumor grade and surgical microscopic and radiological evidence of the total tumor resection.

In conclusion, characteristic pineal apoplectic symptomatology, either spontaneous or induced, is a rare event but requires an expeditious clinical response, notwithstanding the pathological entities and precipitating factors.
